# Age-Related Changes in Sensorimotor Temporal Binding

**DOI:** 10.3389/fnhum.2017.00500

**Published:** 2017-10-12

**Authors:** Tiziana Vercillo, Carlos Carrasco, Fang Jiang

**Affiliations:** Department of Psychology, University of Nevada, Reno, NV, United States

**Keywords:** sensorimotor, ageing (aging), recalibration, adaptation, temporal sensitivity

## Abstract

The causal relationship between a voluntary movement and a sensory event is crucial for experiencing agency. Sensory events must occur within a certain delay from a voluntary movement to be perceived as self-generated. Therefore, temporal sensitivity, i.e., the ability to discriminate temporal asynchronies between motor and sensory events, is important for sensorimotor binding. Moreover, differences in the physical propagation of external stimuli can sometimes challenge sensorimotor binding, generating illusory asynchrony. To overcome this problem, the brain adjusts the perceptual timing of sensory and motor events. This mechanism, named sensorimotor recalibration, helps keeping causality judgments accurate. As humans age, the broad decline in sensory and motor processing may reduce temporal sensitivity, and compromise sensorimotor recalibration. In the current study, we investigated the effect of aging on sensorimotor temporal binding by measuring changes in both temporal sensitivity and recalibration. Young and elderly adults were exposed to a prolonged physical delay between a voluntary movement (a keypress) and its perceptual consequence (a visual stimulus). Before and after this exposure, participants performed a sensorimotor temporal order judgment (TOJ) task. As expected, elderly adults showed reduced sensorimotor recalibration and sensitivity as compared to young adults, suggesting that aging affects sensorimotor temporal binding.

## Introduction

Agency is central to human life as it embraces the conscious experience of changing the external world through behavior. Such mental phenomenon is inherently associated with causality judgments and sensorimotor temporal binding (David et al., 2008). Whether it is determining if a twig snapping is caused by one’s own footstep or by the movement of a predator, or learning to play a video game, the temporal binding of actions and sensory events is an integral part in defining the sense of agency (Haggard and Chambon, 2012).

Causality assessments require the fine ability to discriminate the temporal order between motor and sensory events, i.e., sensorimotor temporal sensitivity. A common notion of causality is that only sensory events that directly follow the onset of a voluntary movement are considered consequences of our own actions rather than the effects of external agents (Blakemore et al., 1999; Moore et al., 2009). Therefore, motor and sensory events that occur within a certain temporal window are usually bound together. On the other side, unrelated information that violate the notion of causality are segregated (Blakemore et al., 1999; Moore et al., 2009; Moore and Fletcher, 2012). In this respect, the perceived temporal relationship between motor and sensory events represents a major factor for causality judgments and sensorimotor binding.

The subjective experience of agency is regulated by the synergic activity of the motor and the sensory systems that process and integrate information from action planning and execution with sensory inputs (David et al., 2008; Moore and Fletcher, 2012). Sensorimotor binding yet must account for differences in the temporal processing within sensory pathways and between sensory and motor pathways (Eagleman et al., 2005). For instance, while motor and proprioceptive inputs are generated and processed within the body, visual and auditory inputs typically travel through the environment to reach the brain. The mechanism of sensorimotor recalibration reduces misleading delays and, in accordance with prior knowledge and/or recent sensory history, regulates the perceptual timing of motor and sensory events keeping causality judgments accurate (Eagleman and Holcombe, 2002; Stetson et al., 2006; Eagleman, 2008; Heron et al., 2009; Sugano et al., 2010; Keetels and Vroomen, 2012). Even a brief exposure to a temporal delay between a simple motor act and its sensory consequences can trigger sensorimotor recalibration (Stetson et al., 2006). The physical delay is wiped out, moving the two events close together.

Sensorimotor abilities are immature in children, develop throughout adolescence and reach a plateau in early adulthood (Vercillo et al., 2014). However, the aging process causes gradual losses to the sensory (Warren et al., 1978; Burton et al., 1993; Spear, 1993; Vesco et al., 1993; Spear et al., 1994; Sturr et al., 1997; Chisolm et al., 2003; Clinard et al., 2010) and the motor system (Erim et al., 1999; Seidler et al., 2010). Even older adults who are free from cognitive impairment still show signs of sensory and motor decline and poor spatial sensorimotor adaptation (Bock and Schneider, 2002; Bock, 2005). More recent works also suggest that temporal sensitivity declines with age, as elderly individuals showed a great difficulty in detecting audiovisual asynchrony (Chan et al., 2014; Stevenson et al., 2017). Moreover, there seems to be indications of a slowing of the internal clock with age indicated by results from unpaced tapping tasks (Turgeon et al., 2011; Turgeon and Wing, 2012). Whether the decline in sensory and motor functions as well as in the sensitivity of temporal perception affects sensorimotor temporal binding is nevertheless still unclear.

In the current study, we investigated sensorimotor temporal binding in young and elderly individuals by measuring: (1) sensorimotor temporal sensitivity, as the ability to discriminate the temporal relationship between motor and sensory events; (2) sensorimotor recalibration, as the ability to adjust the timing of motor and sensory events after adaptation to a sensorimotor asynchrony. Participants were tested in a task requiring the discrimination of the temporal order between a motor and a sensory event. The task was performed before and after the exposure to a temporal delay between their own action and the subsequent sensory consequence. As predicted, we found that aging is associated with a decline in sensorimotor temporal binding.

## Materials and Methods

Ten elderly participants (mean age: 68 ± 0.57, 6 females and 4 males) and 11 young adults (mean age: 31 ± 3 years, 7 females and 4 males) participated in this study. All participants were right handed and had normal or corrected to normal vision. Two elderly participants required the use of hearing aids. All the elderly participants reported no cognitive impairment or neurodegenerative diseases. Younger participants were recruited from the University of Nevada, Reno while elderly individuals were recruited from the Reno (NV) area. All participants read and signed an informed consent before the experiment. The Institutional Review Board at the University of Nevada, Reno approved all experimental protocols.

Experimental procedures and stimuli were adapted from Vercillo et al. (2014). The visual stimulus consisted of a white circle measuring 6° in diameter that was briefly flashed on a gray background for 30 ms. The stimulus was presented on a Display ++ LCD monitor (Cambridge Research System). Motor actions consisted in button presses and were recorded via a CB6 response box that interfaced with Bits#. To cover the sound produced by the button press, participants listened to 65 dB white noise that was delivered through headphones.

During the experiment, participants sat in a dark room at 57 cm from the screen. Sensorimotor synchronization and temporal precision were assessed with a temporal order judgement (TOJ) task. At the beginning of each trial of the TOJ task, a black fixation cross appeared in the center of the screen. After 2 s, the fixation cross disappeared and participants were required to perform a voluntary action by pressing the button on the response box as fast as possible. Participants were trained to perform fast and precise movements. The visual stimulus (a white circle) was displayed at a variable temporal interval from the disappearance of the fixation cross, so that it could have appeared either before or after participants’ button press. At the end of each trial, participants verbally reported whether the circle appeared before or after their button press, thus making a TOJ. Verbal responses and reaction times (RTs) were both recorded in Matlab.

Asynchronies between the visual stimulus and the motor action were partially determined by individual average RTs. First, we calculated individual average RTs, and then we utilized these values to determine stimulus latencies. Latencies were calculated from the timing of the disappearance of the fixation cross and were selected to ensure that the visual stimulus was presented either before or after participants’ button press. Specifically, we tried to establish the following asynchrony values (Stimulus Onset Asynchrony—SOA): ±100 ms, ±80 ms, ±60 ms, ±40 ms, ±20 ms and 0 ms, where negative values indicate that the visual stimulus occurred before the motor action, and positive values indicate that the visual stimulus happened after the motor action. Each latency was repeated 10 times in a constant stimuli algorithm. Note that as individual’s RTs naturally fluctuated around the average, the effective SOA values diverged from those originally selected values. Using this strategy, we were able to deliver the stimulus as early as 300 ms before and 300 ms after the button press.

At the beginning of the experiment, each participant ran a brief training session of 30 trials to familiarize with the TOJ task. During the training, average RTs were fixed at 250 ms. The learning process was facilitated by the occurrence of a feedback to individual responses at the end of each trial. Following the training session, we measured individual average RTs and set stimulus latencies for the TOJ task.

During adaptation, participants were exposed to a 200 ms delay between the motor action and the visual feedback. Participants were instructed to fixate the cross in the center of the screen and press a button at their own will receiving a visual feedback with a delay of 200 ms. We randomly presented catch trials with a different visual stimulus (a dark gray circle) and asked participants to count them and verbally report the number at the end of the adaptation phase. Catch trials were selected from a range of 30–50 by the experimenter with the aim of ensuring participants’ attention and preventing short inter-press intervals.

**Figure 1:**
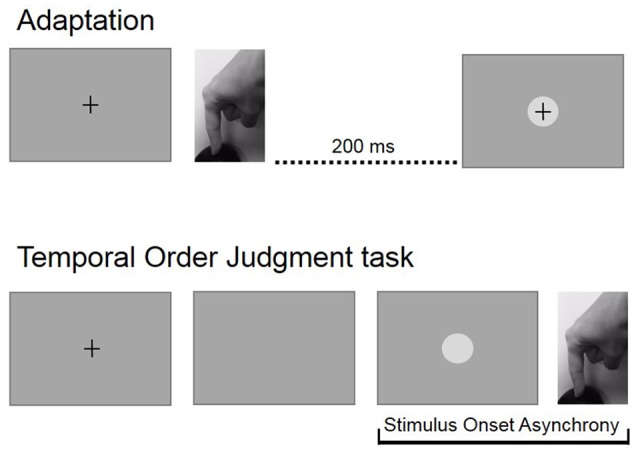
Experimental procedures. In the adaptation block, participants pressed a button at the time of their own will and received a visual feedback with a temporal delay of 200 ms. During the temporal order judgment (TOJ) block, participants had to press a button immediately after the disappearing of the fixation cross and then estimate the temporal relation between their action and a visual stimulus that was displayed with a variable asynchrony.

Participants performed two experimental blocks: a baseline block, consisted of 110 TOJ trials and an adaption blocks that consisted of 100 adaptation trials followed by 110 TOJ trials. The baseline block was performed first. After the baseline block, individual average RTs were recalculated and updated for the adaptation block. Figure 1 shows experimental procedures.

For each experimental block, the proportion of trials where the visual stimulus was perceived as happening after the motor action was computed for each effective SOA value and fitted with cumulative Gaussian functions. The two parameters of the Gaussian (mean and standard deviation) were estimated using the maximum likelihood method (Finney, 1947). The mean value represents the point of subjective simultaneity (PSS), while the standard deviation represents the just noticeable difference (JND).

The PSS represents the SOA value at which the delay between the motor and the sensory event is indiscernible for the participant. The PSS represents a non-discrimination point and is defined as the 50% point of the psychometric function. More in general, the PSS represent the perceived simultaneity between the motor action and the visual stimulus. A PSS equal to 0 indicates that motor and sensory events are perceived as synchronous when they physically occur at the same time. PSS values different from 0 denote a perceptual bias. Specifically, a positive PSS value indicates that the sensory and the motor events have been moved closer in time. In this case, the perceived synchrony befalls when the visual stimulus is delayed. After measuring PSS values in the adaptation and the baseline condition, we assessed the recalibration effect by subtracting these two values. A positive effect denotes sensorimotor recalibration: a temporal compression between the sensory and the motor events.

As an index of sensorimotor temporal sensitivity, we measured the JND, the standard deviation of the function. The JND represent the minimum temporal delay between motor action and visual feedback to produce a JND in temporal perception. Standard errors for the PSS and JND estimates were obtained with a bootstrap procedure (Efron and Tibshirani, 1993). Trials from the training block were excluded from analysis. We also measured RTs during the TOJ task, calculated as the temporal interval between the disappearance of the fixation cross and the button press.

Sensorimotor recalibration was measured as the difference in the PSS between the adaptation and the baseline condition. Values were calculated individually and then averaged across participants for each group.

Data were analyzed with two-tailed *t*-tests, with the Analysis of Variance (ANOVA) and with Linear Regression Analysis using SPSS 22 software (SPSS, Inc., Chicago, IL, USA).

## Results

**Figure 2:**
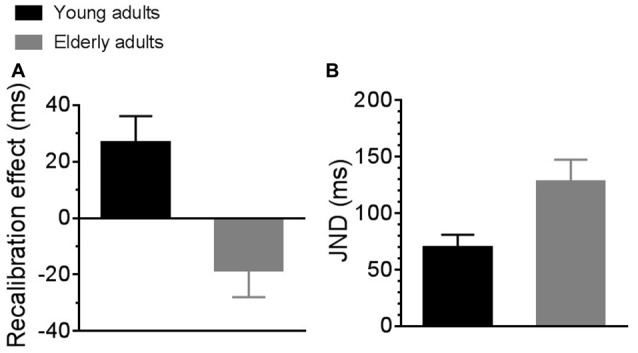
Average sensorimotor recalibration effect **(A)** and just noticeable differences (JNDs; **B**) for young (black bars) and elderly (gray bars) participants.

Differences in sensorimotor temporal binding between the two groups of participants are reported in Figure 2. The left panel of the figure shows the recalibration effect for young (black bar) and elderly (gray bar) adults. Young adults showed a significantly higher recalibration effect as compared to elderly adults (2-tailed, independent samples *t*-test with Bonferroni correction for multiple comparison; *t*_19_ = 3.45, *p* = 0.003). In particular, young adults showed a positive recalibration effect greater than 0 (2-tailed, one sample *t*-test with Bonferroni correction for multiple comparison; *t*_10_ = 2.9, *p* = 0.01) while elderly adults showed a tendency toward a negative effect of recalibration that nevertheless was not significant (2-tailed, one sample *t*-test with Bonferroni correction for multiple comparison; *t*_9_ = −1.99, *p* = 0.07).

The right panel of the figure shows average JNDs for young (black bar) and elderly (gray bar) adults. JNDs for elderly participants were significantly higher than for young participants, suggesting poorer sensorimotor temporal sensitivity in the elderly group. A repeated measure ANOVA (experimental condition * group) confirmed an effect of group (*F*_(1,19)_ = 7.66, *p* = 0.01, partial *η*^2^ = 0.28) but no effect of experimental condition, as JNDs were similar between baseline and adaptation condition. For this reason, in the figure we reported JNDs averaged across experimental conditions.

**Figure 3:**
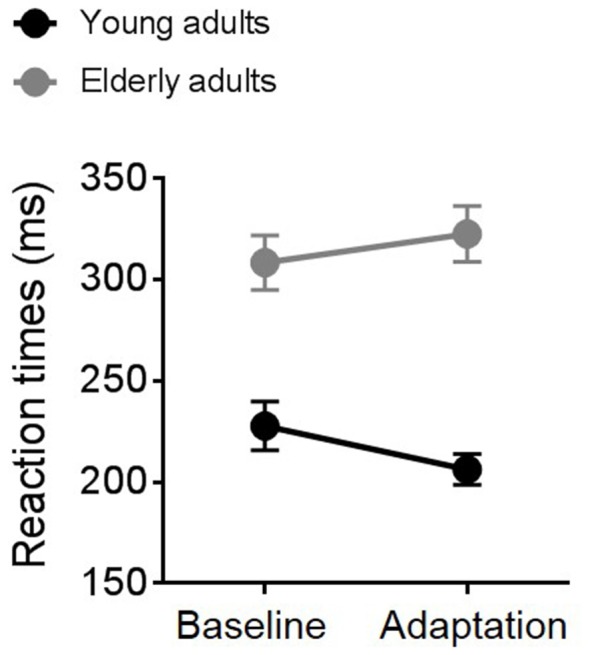
Average reaction times (RTs) for young (black symbols and line) and elderly (gray symbols and line) participants in the baseline and the adaptation condition.

Average RTs measured during the TOJ task in the adaptation and the baseline condition for the two groups of participants are shown in Figure 3. A repeated measure ANOVA revealed an interaction between experimental condition and group (*F*_(1,19)_ = 5.14, *p* = 0.03, partial *η*^2^ = 0.21) and a significant effect of group (*F*_(1,19)_ = 11.33, *p* = 0.003, partial *η*^2^ = 0.37). Overall, elderly individuals (gray symbols and line) reacted slower than young participants (black symbols and line) to the go signal. Interestingly, elderly and young adults showed an opposite pattern of responses across the two experimental conditions. While elderly participants slowed down motor responses in the second block (the adaptation block), young participants showed faster RTs during adaptation.

**Figure 4:**
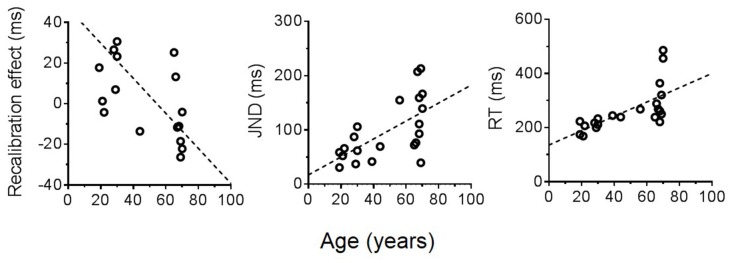
Age-related changes of the recalibration effect, JNDs and RTs.

We found a negative correlation between recalibration effect and age (*R*^2^ = 0.23, *p* = 0.02). Only young adults exhibited a positive recalibration effect. Elderly participants did not compress the temporal interval between action and sensory event, and sometimes even showed an opposite pattern of adaptation. Sensorimotor temporal sensitivity decreased with age, considering that individual JNDs became higher as the age increased (*R*^2^ = 0.38, *p* < 0.005). Similar to JNDs, RTs substantially increased with age (*R*^2^ = 0.44, *p* < 0.001). In Figure 4, we reported changes in the recalibration effect, JNDs and RTs associated with age. This figure shows individual data for all the participants (*n* = 21) fitted with a linear regression model.

## Discussion

Agency refers to the experience of controlling our own actions and producing consequences in the outside world (Haggard and Chambon, 2012). In everyday life, we have dealings with a continuous stream of information and sometimes distinguishing sensory events that we produce from those effected by others may be challenging. Sensorimotor binding (the ability of grouping together actions and sensory consequences that are linked by a causal relationship) strongly depends on temporal sensitivity and recalibration. The brain must understand temporal relations between motor and sensory events and adjust these perceptual judgments in case of misleading delays. In the current study, we investigated changes in sensorimotor temporal binding and found a reduced ability of grouping sensory and motor events caused by alterations in temporal sensitivity and sensorimotor recalibration.

It is well documented that aging induces a general slowing of cognitive processing that affects the behavioral performance of elderly adults in several tasks (Cerella, 1985; Birren and Fisher, 1995). However, results from this study together with previous researches on multisensory and sensorimotor integration (Wolpe et al., 2016; Stevenson et al., 2017), support the notion that age-related differences in sensorimotor processing could not be explained by general cognitive slowing. The poor sensorimotor binding might rather be attributable to a decrease in temporal sensitivity and a decline of sensory and motor processing. Indeed, older adults can hardly separate auditory and visual signals in time, as their temporal window of integration widens (Laurienti et al., 2006; Setti et al., 2011; Mozolic et al., 2012; Bedard and Barnett-Cowan, 2016) and these temporal deficits might be related to a reduced sensitivity or acuity in the sensory systems (Schmolesky et al., 2000; Ostroff et al., 2003; Liu and Yan, 2007; Shaffer and Harrison, 2007; Mozolic et al., 2012).

In the course of healthy aging, sensory and motor abilities decline and a linear reduction of the volume in association cortices and white matter becomes apparent (Raz et al., 2005). The combination of sensory information into intentional motor response deteriorates with age generating balance impairments (Maki and McIlroy, 1996) and increasing the risk of falls (Tinetti et al., 1988). Similarly, in our study we found that temporal sensorimotor binding declines with age. Elderly participants experienced troubles in representing causal relationship between motor and sensory events. The lower sensitivity for sensorimotor asynchrony may represent the source of different experiences of agency between young and elderly adults. More importantly, the loss of sensorimotor recalibration with age points out a decrease in the flexibility of sensorimotor binding and a poor adaptability to environmental conditions for the elderly population.

In agreement with our results, a recent study by Wolpe et al. (2016) showed increased sensorimotor attenuation, a reduction in the perceived intensity of sensations when they are induced by a voluntary action, in elderly as compared to young adults. The effect was related to differences between elderly and young adults in the volume and the connectivity of the pre-supplementary motor area with prefrontal and striatal regions. Following the loss of sensory precision, predictive signals generated by voluntary movements may be over-weighted, and sensory information down-weighted. Authors suggested that the increased attenuation in the elderly population might represent a compensatory mechanism to preserve the sense of agency. Indeed, the increased noise within the sensory and the motor pathways might challenge the ability to discriminate between sensory events that are the consequences of our own actions rather than the effects of external agents. Similarly, previous studies found that the ability to integrate multiple sensory signals into a perceptual gestalt declines with age (Stevenson et al., 2017) and that aging impacts on unisensory (Gelfand et al., 1980; Robin and Royer, 1989) and multisensory (Chan et al., 2014) temporal processing. Together, these findings suggest that the binding of sensory and motor input changes across the lifespan, with elderly individuals showing deficit in ordinary tasks such as speech production and perception, and spatial navigation.

Age-related reduction in attentive efficiency (Madden, 2007) and in selective inhibition of irrelevant stimuli (McDowd and Oseas-Kreger, 1991; Chao and Knight, 1997) might also be critical for the lack of sensorimotor recalibration and the poor temporal sensitivity that we found in elderly participants. As described in Figure 3, RTs for elderly participants became slower in the second experimental block, the adaptation block, suggesting fatigue and a possible drop in attentional resources. On the contrary, RTs of young participants became faster, suggesting an automatization of the task. As revealed by a previous study, top-down attention can modulate the ability to adjust temporal judgments based on recent sensory history (Heron et al., 2010) and therefore the involvement of this higher cognitive process deserves further investigations.

Understanding the nature of age-related changes in sensorimotor binding and their connections with a more general decline in cognitive functions is critical for developing interventions aimed to improve the quality of life of elderly individuals. Future researches should clarify the linkage between age-related deficit in sensorimotor and multisensory temporal binding and assess whether they are in some way modulated by impairment in higher cognitive processes.

## Author Contributions

TV and FJ: conception and design of study, revising the manuscript critically for important intellectual content. TV and CC: acquisition of data, drafting the manuscript. TV: analysis and/or interpretation of data.

## Conflict of Interest Statement

The authors declare that the research was conducted in the absence of any commercial or financial relationships that could be construed as a potential conflict of interest.
